# Occupational burnout and job satisfaction among nurses in designated infectious disease hospitals: a correlational study

**DOI:** 10.3389/fpubh.2026.1744624

**Published:** 2026-03-20

**Authors:** Wenjie Liu, Sheng Li, Jinyu Wang, Fei Wang

**Affiliations:** 1 School of Public Health, Gansu University of Chinese Medicine, Lanzhou, China; 2 The No.2 People’s Hospital of Lanzhou, Lanzhou, China; 3 School of Public Health, Lanzhou University, Lanzhou, China

**Keywords:** designated hospital for infectious diseases, job satisfaction, non-permanent staff, nurses, occupational burnout

## Abstract

**Objective:**

The purpose of this study is to determine the factors that influence occupational burnout and job satisfaction in nurses working at designated infectious disease hospitals in Gansu Province, as well as to look into the relationship between these two variables.

**Method:**

7,200 nurses from designated infectious disease hospitals in Gansu Province were chosen as study participants for a cross-sectional survey using a convenience sample technique in March and April of 2024. Burnout levels were measured using the Maslach Burnout Inventory, and job satisfaction was measured using the Minnesota Satisfaction Questionnaire (MSQ). The Wilcoxon Z and Kruskal-Wallis H tests were used to compare occupational burnout and job satisfaction among nurses with different demographics. Multiple linear regression was utilized to determine the factors impacting job satisfaction, and Spearman’s correlation analysis was utilized to investigate the link between the two variables.

**Results:**

7,200 questionnaires in total were gathered for this investigation. 6,963 valid questionnaires were obtained after 237 invalid responses were eliminated, resulting in a valid response rate of 96.71%. In Gansu Province, 6,963 nurses working in designated infectious disease hospitals reported an overall burnout score of 64 (56, 72) and a job satisfaction score of 73 (61, 80). The results of the study show that occupational burnout and nurses’ job satisfaction at hospitals designated for infectious diseases are significantly correlated negatively (*r* = −0.151, *p* < 0.01). Monthly salary, years of service, administrative position, and occupational burnout are the main factors impacting job satisfaction, according to multiple linear regression analysis (all *p* < 0.05).

**Conclusion:**

Overall job satisfaction is high among nurses at Gansu Province’s designated infectious disease hospitals, despite modest levels of occupational burnout. One major aspect affecting job happiness is occupational burnout. It is advised that healthcare workers’ occupational burnout be reduced and their job satisfaction increased by taking steps including enhancing psychological interventions, improving promotion procedures, and optimizing remuneration systems.

## 1 Introduction

Numerous new and unexpected infectious diseases continue to arise regularly and at high rates in the post-pandemic age. In designated hospitals for infectious diseases, nurses still have to deal with issues including occupational exposure, standard emergency response protocols, and cramped workspaces. Occupational burnout is still a major problem as a result of ongoing high psychological stress levels brought on by both physical and mental strain (1). The term “occupational burnout” describes a complicated psychological state that people who endure extended high-intensity work stress suffer. It is marked by emotional tiredness, depersonalization, and diminishing personal accomplishment. It is a common symptom of work-related stress that has gone untreated for a long time (2). Nurses’ pay, job management, and career development are undergoing structural changes as China’s healthcare reform moves forward. The high-risk nature of their jobs and the particular demands of their duties present extra difficulties for this group, such as heavier workloads and a lack of acknowledgment of risk contributions in promotions. These elements undermine their professional identity and job happiness, exacerbating occupational burnout even further (3–5). Based on their professional expectations and real work experience, job satisfaction is the term used to describe an individual’s overall favorable assessment of elements including job content, pay and perks, career advancement, and organizational support. It is a fundamental measure of psychological health and occupational fit (6). A crucial theoretical framework for examining the previously mentioned problems is offered by the Job Demands-Resources (JD-R) hypothesis. According to this idea, there are two main categories of job conditions: job demands and job resources. The former describes circumstances like high-intensity workloads and occupational exposure that cause stress and resource depletion; persistent overload in these circumstances directly results in the weariness and exhaustion that characterize burnout. As vital buffers for improving job satisfaction and lowering burnout, the latter refers to supporting circumstances like equitable pay and organizational assistance that reduce stress and restock resources. This theory’s central claim is that when job expectations and resources are out of balance, burnout and job satisfaction rise and fall at the same time. This pattern closely resembles the work environment of infectious disease nurses (7). Occupational burnout among infectious disease nurses, who are the backbone of public health prevention and control, raises the risk of cross-infection in addition to compromising the standard of infectious disease diagnosis, treatment, and care. Additionally, it can result in the departure of key staff, endangering the public health emergency response system’s stability (8). Preventing and reducing significant public health threats is a strategic endeavor that should never be overlooked, according to the Healthy China 2030 Plan (9). Infectious disease hospitals, a vital component of the public health system, regard their nurses as having a direct impact on the standard and safety of medical care in addition to their dual duties of normal care and emergency response (10, 11). Team stability and service quality are significantly impacted by job burnout and job satisfaction, which together represent a person’s psychological condition and professional identity (12). It has particular importance for public health since it also pertains to risk response capabilities and the efficacy of preventive and control.

Burnout and job satisfaction among nurses are currently negatively correlated, a finding that has been repeatedly confirmed by numerous empirical investigations (13, 14). However, the majority of pertinent research has been done in the developed eastern regions and has concentrated on general hospitals or primary healthcare facilities. Few studies have addressed particular aspects like occupational exposure and environmental confinement, and research targeting nurses in designated infectious disease hospitals infectious disease hospitals in poor regions of Northwest China is still lacking. Furthermore, a variety of factors, such as personal traits, job features, and risk mitigation strategies, all have an impact on job satisfaction levels (15). Improving job happiness is especially important for nurses at designated infectious disease hospitals who work long hours under extreme pressure and significant danger. In addition to successfully reducing the symptoms of occupational burnout, such as depersonalization and emotional tiredness, it also fosters better performance in terms of patient diagnosis and treatment quality, the effectiveness of emergency response during outbreaks, and the recognition of professional value. Consequently, this offers a strong basis for the steady functioning of the public health system (16). Accordingly, nurses in hospitals in Gansu Province that have been recognized as infectious disease hospitals are the primary focus of this study. Thoroughly evaluate the occupational burnout and job satisfaction that they are now experiencing, investigate the factors that affect nurses’ job happiness, and close any study gaps pertaining to certain groups and situations. To offer a solid scientific foundation for improving specific HR policies, maintaining the talent pool, and guaranteeing the public health system runs smoothly.

## 2 Subjects and methods

### 2.1 Research subjects

This study, conducted in March and April of 2024, covered all designated treatment units for comparable infectious diseases in Gansu Province, China, which accounted for 100% of the province’s pertinent medical facilities.7,200 nurses from 88 approved HIV/AIDS and TB treatment centers in Gansu Province, China, were questioned using a convenience sampling technique. The sample is entirely representative. Every participant in this study was chosen from hospitals that specialize in infectious diseases. Only internal departments and roles within these designated infectious disease hospitals are referred to by the classifications used in the text, such as clinical departments, emergency departments, medical technology positions, and administrative departments. Nursing professionals who have earned a Nurse Practice Certificate, finished a valid practice registration, and are presently working in the nursing field are expressly referred to as “nurses” in this study. Within the nursing sequence, this encompasses general healthcare professionals (registered nurses) and nursing management roles (department directors/deputy directors, department head nurses/head nurses). None of them are categorized as administrative staff outside of the nursing sequence or other specialized healthcare professionals, and they are all currently employed inside the nursing sequence and possess valid registered nurse credentials. The research subjects are well-defined and adhere to the rules of publication ethics. Requirements for Study Participant Inclusion: (1) A minimum of 1 year’s work experience, encompassing both contract and permanent employees; (2) Registered nurses who are currently licensed;(3) Lack of a history of serious physical conditions (as determined by medical facilities, necessitating long-term medication or hospitalization, and substantially reducing the ability to engage in physical activity or daily work efficiency, such as cancerous tumors, heart or brain trauma, chronic organ failure, etc.); (4) No recent psychological intervention or psychiatric pharmaceutical treatment within the last 3 months, as well as no diagnosis of anxiety disorders, depression, or other mental health illnesses from a qualified professional organization; (5) Informed consent and willingness to take part in the study. Exclusion criteria include: (1) trainee nurses; (2) intern nurses; (3) rehired nurses; (4) nurses who are nursing or on maternity leave; and (5) nurses who have been diagnosed with mental health disorders like depression or anxiety. The Gansu Provincial Tuberculosis Medical Quality Control Center organized this study, which was approved by the Gansu Provincial Health Commission. Informed consent was given by each subject. P-KY20250500025 is the ethical review approval number.

### 2.2 Survey instruments

#### 2.2.1 General information questionnaire

A self-designed questionnaire was used to collect sociodemographic characteristics of research subjects, including current workplace, gender, age, department, position, professional title, highest level of education, marital status, years of service, and monthly income. Among these variables, hospital affiliation, institution level, employment status, administrative position, and professional title involve classification standards unique to China’s healthcare system. To avoid misinterpretation, the core classification dimensions of the aforementioned variables are explicitly defined as follows: (1) Hospital Affiliation: The administrative hierarchy of medical institutions, including provincial-level, municipal-level, and district/county-level hospitals, and township health centers/community health service centers serving grassroots populations. (2) Level: China’s official comprehensive strength rating system for medical institutions. Grade III Class A is the highest domestic rating, representing top-tier medical technology and management standards; Grade III Class B, Grade II Class A, and Grade II Class B are subsequent tiers within Grade III and Grade II hospitals, respectively. (3) Employment Status: The employment type of medical institution personnel. “Permanent Staff” are positions in government-approved staffing quotas with stable career security and benefits; “Contract Staff” are non-quota positions via labor contracts with flexible arrangements. (4) Administrative Positions: Internal management roles in medical institutions, including Department Director/Deputy Director (overseeing department administration and operations), Ward Nurse Manager/Nurse Manager (managing nursing units), and non-administrative medical staff. (5) Professional Titles: The hierarchical classification of professional technical levels in healthcare, ranked by expertise from lowest to highest: Junior, Intermediate, Associate Senior, and Senior (corresponding to entry-level, experienced, high-level professional, and top-tier technical proficiency). Others are individuals ineligible for medical professional title evaluation.

#### 2.2.2 Occupational burnout assessment

Huang Xuefei translated the Burnout Inventory into Chinese, and this study used that version (2). This scale’s initial iteration was acquired from Psychometrics Canada Ltd. Standard cross-cultural adaptation protocols were closely adhered to in the Chinese adaption process: First, the first translations were independently performed by two psychology graduate students. The two manuscripts were compared and examined to create a preliminary version. A revised version was then produced after medical and nursing professionals were invited to take part in a collaborative conversation to edit and improve the original document. Based on this basis, a pilot research with a small sample of nurses was used to validate the scale’s performance; the results showed a validity coefficient of 0.92. After being examined by regional specialists, the Chinese translation was finally sent to the Canadian Psychological Testing Institute and accepted. The Maslach Burnout Inventory is currently the most popular and well-known standardized measurement tool for occupational burnout in the world. The international academic community has thoroughly confirmed the Maslach Burnout Inventory’s application and authority. The MBI family of instruments has shown outstanding psychometric qualities in empirical research carried out overseas. Not only did the test–retest reliability and Cronbach’s alpha coefficient reach optimal levels, but it also passed rigorous testing for convergent validity and discriminant validity. It accurately captures the three-dimensional structure of burnout and successfully differentiates it from comparable notions like work stress and emotional weariness (17). Li et al. (18) have verified that the MBI series of scales exhibits strong validity and reliability among the Chinese population for localized application scenarios in China. Its dimensional structure is very similar to the ways that occupational burnout manifests itself in China’s working population. Additionally, a review by domestic scholars based on pertinent literature from 2002 to 2022 further validated the applicability of the MBI for high-stress occupational groups like nursing, confirming its suitability for large-scale surveys among time-constrained professional groups like clinical nurses (12). There are 22 items total on the scale, which are divided into three categories: low personal accomplishment, depersonalization, and emotional weariness. There are nine items in the emotional tiredness dimension, five in the depersonalization dimension, and eight in the poor personal accomplishment dimension. With a scoring range of 0 to 6, the scale uses a 7-point Likert self-assessment. The matching requirements are: 0 means “never,” 1 means “many times a year,” and 2 means “roughly once a month.” 3 = “A few times per month” 4 = “Roughly once per week” 5 = “A few times a week” and 6 = “Daily.” According to the grading guidelines, every item falling under the categories of dehumanization and emotional weariness receives a favorable score. In other words, the associated dimension score increases with the item’s self-reported score. When an item’s self-rated score is higher, the related dimension score is lower. This is the case for all items under the low personal achievement dimension. The official scale specifications are scrupulously followed when scoring. Using the aforementioned positive and negative scoring guidelines in conjunction with the results of the self-assessment, items are directly scored from 0 to 6. The total of all the objects in a given dimension is its score. Nine elements add up to the Emotional Exhaustion dimension score, five items add up to the Dehumanization dimension score, and eight items add up to the Low Personal Accomplishment dimension score. The Burnout Scale’s overall score, which ranges from 0 to 132 points, is the sum of the three dimension values. Each dimension’s score as well as the overall score were used to calculate the results. More severe occupational burnout is indicated by lower ratings on the low personal accomplishment dimension and higher scores on the emotional tiredness and depersonalization aspects. On the other hand, milder occupational burnout is indicated by lower scores on these parameters. According to conventional interpretation guidelines for nursing populations, this study classifies burnout severity based on the MBI total score: scores <50 indicate low burnout, scores 50–69 suggest moderate burnout, and scores ≥70 indicate high burnout. The scale and its dimensions in this study have Cronbach’s *α* coefficients of 0.893, 0.931, 0.866, and 0.903, respectively, showing good reliability.

#### 2.2.3 Job satisfaction survey

Weiss et al. (6) created the Minnesota Satisfaction Questionnaire (MSQ) Short Form, a traditional instrument that is used extensively throughout the world to assess job satisfaction. Consists of a short-form and a long-form version. Because of their lengthy responses and intricate methods, long-form surveys often add to the stress of the respondent. Additionally, because of respondent weariness, negligent marking, or other circumstances, they could bring bias into research conclusions. As a result, its use is restricted in situations involving time-sensitive research or extensive surveys. The short-form scale optimizes item design while maintaining essential assessment features. This version has superior psychometric qualities in addition to successfully avoiding the verbosity that long-form scales inherently have. Overall, the Cronbach’s *α* coefficient was 0.94, which indicates steady, trustworthy measurement results and strong internal consistency dependability of the scale (19). This study used a short-form scale. Extrinsic factors (elements that hinder job satisfaction, such as supervision, compensation, policies, working conditions, interpersonal relationships, and safety) and intrinsic factors (elements that promote job satisfaction, such as opportunities for promotion and growth, recognition, responsibility, and achievement) are typically taken into account when evaluating job satisfaction. There are 20 items on this scale. Extrinsic satisfaction indicators are evaluated in items 5, 6, 12, 13, 14, and 19; intrinsic satisfaction indicators are evaluated in items 1–4, 7–11, 15, 16, and 20; and overall job satisfaction is determined in items 17 and 18. With response options ranging from “Very Dissatisfied” to “Very Satisfied,” which equate to scores of 1 to 5, the measure uses a 5-point Likert rating. Every item has a positive score, indicating that improved performance in each domain and general job satisfaction are indicated by higher self-reported scores. The official scale specifications are scrupulously followed when scoring. Based on their self-assessment results and the previously specified positive rating guidelines, entries are immediately evaluated from 1 to 5. The total of all the scores for the entries in a given dimension is the score for that dimension. The scores of the corresponding six items are added together to determine the external satisfaction dimension score. The scores of the corresponding 11 items are added up to determine the internal satisfaction dimension score. The scores of the two equivalent items are added together to determine the overall job satisfaction score. The sum of the scores for each of the 20 items makes up the total scale score, which ranges from 20 to 100 points. Higher scores indicate more job satisfaction, and the findings are based on the sum of the scores for each dimension (20). The scale and its dimensions in this study have Cronbach’s *α* coefficients of 0.966, 0.944, 0.930, and 0.813, respectively, showing strong reliability.

#### 2.2.4 Survey procedures and quality control

The Burnout Scale, the Job Satisfaction Scale, and general demographic data make up the three components of this study questionnaire. The “QuestionStar” platform was used to create an electronic questionnaire. Data was gathered utilizing an online convenience sample technique following expert evaluation, which included two clinical management professionals and three public health academics, which validated the questionnaire’s logical consistency and scientific validity. Prior to the survey, provide investigators with standardized training to help them understand the requirements for completing the questionnaire. During the survey, establish time limitations for responses (minimum response time > 5 min), logical validation, and mandatory fields to avoid invalid questions. Two members of the research team will cross-check the data after the survey is over, eliminating questionnaires that have unusually short completion times, patterned responses (such choosing the same answer for every question), or important information missing. A total of 7,200 questionnaires were collected for this survey. After screening according to the aforementioned criteria, 6,963 valid questionnaires were ultimately included, yielding a valid response rate of 96.7%.

#### 2.2.5 Statistical analysis

Data were organized using WPS 12.1 software and statistically analyzed using SPSS 26.0 software. The Shapiro–Wilk test was used to assess the normality of quantitative data. Since the quantitative data did not follow a normal distribution, they were described using the median and interquartile range [Md(P_25_, P_75_)]. Count data were described using frequency and percentage. Intergroup differences were assessed using the Kruskal–Wallis H test or the Mann–Whitney U test; Spearman’s correlation analysis was employed to examine the relationship between job satisfaction and occupational burnout. Using multiple linear regression analysis to investigate the factors influencing job satisfaction. Using job satisfaction total score as the dependent variable, variables statistically significant at the *α* = 0.05 level of significance were included in the univariate analysis. Regression model assumptions were checked before modeling: residual normality was confirmed by residual normality tests; residual scatterplot trends were used to evaluate homoscedasticity; all variables had variance inflation factors (VIF) < 5, which indicates that there is no multicollinearity; and there was a linear trend between the independent and dependent variables. Independent influencing factors were examined following stepwise selection, R2 goodness-of-fit evaluation, and collinearity diagnostics. The significance level for all statistical tests was set at *α* = 0.05.

## 3 Results

### 3.1 Basic characteristics of study participants

This study employed a convenience sampling method, selecting nurses from designated infectious disease hospitals as research subjects, with a total of 6,963 valid samples included. Among the 6,963 healthcare workers, nearly 70% were employed at tertiary hospitals, while 68.3% worked at district-level hospitals. The majority were female, totaling 6,824 individuals (98.0%); ages were predominantly concentrated between 20 and 40 years old (over 87%); among the departments, 5,771 staff members are in clinical departments (accounting for 82.9%); in terms of professional titles, 4,161 staff members hold junior-level positions (accounting for 59.8%); in administrative roles, 6,274 staff members are general medical personnel (accounting for 90.1%); there are 5,706 non-permanent staff, accounting for 81.9%; the largest group has 11–20 years of service, totaling 2,357 individuals (36.4%); and over 94% have monthly incomes below 6,000 yuan. The majority held bachelor’s degrees or higher, totaling 4,999 individuals (71.8%); married individuals constituted the largest marital group, numbering 5,786 (83.1%); and 62.4% of nurses reported having 1–2 hobbies or interests. See Table 1.

**Table 1:** Basic survey data on nurses in designated infectious disease hospitals [*n*(%)].

Characteristic	Number surveyed (%)	Characteristic	Number surveyed (%)
Institution level		Hospital affiliation	
Tertiary grade A	2,376 (34.1)	Provincial	247 (3.5)
Grade B level III	2,453 (35.2)	Municipal	1957 (28.1)
Grade 2 class A	1949 (28.0)	District/county level	4,755 (68.3)
Second-class grade B	185 (2.7)	Township health centers/community service centers	4 (0.1)
Gender		Nature of work	
Male	139 (2.0)	Permanent staff	1,257 (18.1)
Female	6,824 (98.0%)	Non-permanent staff	5,706 (81.9)
Age (years)		Department	
20–30	2,697 (38.7)	Emergency department	376 (5.4)
31–40	3,302 (47.2)	Clinical departments	5,771 (82.9)
41–50	759 (10.9)	Medical technology departments	613 (8.8)
≥51	205 (2.9)	Administrative departments	203 (2.9)
Monthly income		Administrative position	
Below 3,000	3,144 (45.2)	Department director/deputy director	36 (0.5)
3,000–6,000	3,439 (49.4)	Head nurse/nurse manager	653 (9.4)
Over 6,000	380 (5.5)	General medical staff	6,274 (90.1)
Years of work experience (years)		Professional title	
≤5	1,741 (25.0)	Junior	4,161 (59.8)
6–10	1916 (27.5)	Intermediate	2,155 (30.9)
11–20	2,357 (36.4)	Associate senior	397 (5.7)
≥21	769 (11.0)	Full professor	20 (0.3)
Marital status		Other	230 (3.3)
Unmarried	1,084 (15.6)	Highest education level	
Married	5,786 (83.1)	College degree or below	1962 (28.2)
Divorced or widowed	93 (1.3)	Bachelor’s degree and above	4,999 (71.8)
Hobbies and interests		Number of children	
None	1998 (28.7)	No children	1,649 (23.7)
1–2 items	4,343 (62.4)	One child	2,644 (38.0)
Many	622 (8.9)	Second child and beyond	2,670 (38.3)

General medical staff refers to regular clinical nurses (non-management positions, licensed registered nurses).

### 3.2 Job satisfaction survey results

The study found that 6,963 nurses at designated infectious disease hospitals had an overall job satisfaction score of 73 (61, 80), an intrinsic satisfaction score of 44 (21, 22), an extrinsic satisfaction score of 21 (18, 23), and an overall satisfaction score of 8 (6, 8), with the intrinsic satisfaction score being relatively high. When examining individual sub-items, the satisfaction level for “maintaining a consistently busy state” within intrinsic satisfaction was relatively low, scoring 3 (3, 4) points. The lowest score for “Alignment of Compensation and Workload” in external satisfaction was 3 (2, 4), and the second-highest score was for “Opportunities for Career Advancement,” scoring 3 (3, 4). Unit level, gender, age, department, professional title, administrative position, job nature, years of service, monthly income, and hobbies were among the categories in which nurses at designated infectious disease hospitals showed statistically significant differences (*p* < 0.05) in their job satisfaction scores. The job satisfaction scores of nursing managers in administrative roles, such as department heads/deputy heads and ward/unit nursing supervisors, were 78 (67.5, 81) and 78 (67, 82), respectively. These scores were significantly higher than the 72 (60, 80) of general medical staff. Job satisfaction showed a slight upward trend with increasing age; regular staff scored 74 (63, 80) points, slightly higher than non-regular staff at 73 (60, 80); Female satisfaction scores averaged 73 (61, 80) points, higher than males’ 67 (59, 79) points; the group with monthly incomes over 6,000 yuan had the highest number at 76 (68, 81); the most significant differences were between administrative departments and those with 1–2 hobbies compared to similar categories. Grade III Class A hospitals scored 74 (61, 80), outperforming hospitals of other grades. See Table 2.

**Table 2:** Job satisfaction scores of nurses in designated infectious disease hospitals by different characteristics [points, Md(P_25_–P_75_)].

Characteristic	Job satisfaction score	Characteristic	Job satisfaction score
Institution level		Hospital affiliation	
Tertiary grade A	74 (61, 80)	Provincial	76 (61, 80)
Grade B level III	72 (60, 80)	Municipal	73 (61, 80)
Grade 2 class A	73 (61, 80)	District/county level	73 (61, 80)
Second-class grade B	72 (60, 80)	Township health centers/community service centers	64 (55, 85)
H	13.032	H	4.067
P	0.005	P	0.254
Gender		Nature of work	
Male	67 (59, 79)	Permanent staff	74 (63, 80)
Female	73 (61, 80)	Non-permanent staff	73 (60, 80)
Z	1.645	Z	1.949
p	0.009	P	<0.001
Age (years)		Department	
20–30	73 (60, 80)	Emergency department	70 (60, 80)
31–40	72 (60, 80)	Clinical departments	73 (61, 80)
41–50	74 (63, 80)	Medical technology departments	72 (60, 80)
≥51	75 (66, 80)	Administrative departments	75 (66, 81)
H	10.879	H	23.116
P	0.012	P	<0.001
Monthly income		Administrative position	
Below 3,000	70 (60, 80)	Department director/deputy director	78 (67.5, 81)
3,000–6,000	75 (63, 80)	Head nurse/nurse manager	78 (67, 82)
Over 6,000	76 (68, 81)	General medical staff	72 (60, 80)
H	155.008	H	61.951
P	<0.001	P	<0.001
Years of work experience (years)		Professional title	
≤5	74 (61, 80)	Junior	74 (61, 80)
6–10	72 (60, 80)	Intermediate	72 (60, 80)
11–20	73 (60, 80)	Associate senior	76 (65, 80)
≥21	74 (64, 80)	Full professor	78 (70.5, 83)
H	20.631	Other	71 (61, 80)
P	<0.001	H	23.643
Marital status		P	<0.001
Unmarried	72 (60, 80)	Highest education level	
Married	73 (61, 80)	College degree or below	73 (61, 80)
Divorced or widowed	70 (60, 79)	Bachelor’s degree and above	73 (61, 80)
H	1.666	Z	0.476
P	0.435	P	0.977
Number of children		Hobbies and interests	
No children	73 (60, 80)	None	69 (60, 80)
One child	74 (61, 80)	1–2 items	75 (63, 80)
Second child and beyond	73 (61, 80)	Many	73 (61, 80)
H	2.035	H	95.415
P	0.362	P	<0.001

### 3.3 Situation of occupational burnout

The total burnout score among 6,963 Nurses at designated infectious disease hospitals was 64 (56, 72) points. The emotional exhaustion dimension scored 24 points (17.35), the depersonalization dimension scored 12 points (9.16), and the reduced personal accomplishment dimension scored 24 points (17.35). Significant differences in burnout scores were observed across various groups: statistically significant differences were found in burnout scores based on age, department, administrative position, job nature, years of service, monthly income, and hobbies (*p* < 0.05). Among these, the comparison of burnout scores across administrative positions revealed that general medical staff scored 65 (56, 72) points, higher than department directors/deputy directors in management roles 60.5 (47.5, 72) points and department head nurses/chief nurses 63 (53, 71) points. Those with a monthly income of 6,000 yuan or more receive the highest score of 70 (60, 80) points. An analysis of burnout scores by department dimension revealed that nurses in administrative departments scored 61 (51, 70) points, ranking lower than emergency departments [64 (56, 71) points], clinical departments [65 (56, 72) points], and medical technology departments [62 (54, 71) points]. Differences in hobbies and interests were most pronounced, with those reporting 1–2 hobbies showing the lowest burnout scores at 64 (54, 71). Nonpermanent staff exhibited slightly higher burnout scores at 65 (56, 72) compared to permanent staff at 64 (54, 72). See Table 3.

**Table 3:** Occupational burnout scores among nurses at designated infectious disease hospitals under different characteristics [points, Md(P_25_–P_75_)].

Characteristic	Burnout score	Characteristic	Burnout score
Institution level		Hospital affiliation	
Tertiary grade A	65 (55, 72)	Provincial	66 (56, 73)
Grade B level III	64 (56, 72)	Municipal	64 (55, 72)
Grade 2 class A	64 (56, 71)	District/County Level	64 (56, 72)
Second-class grade B	66 (57, 72)	Township health centers/community service centers	71 (64, 77)
H	2.287	H	3.968
P	0.515	P	0.265
Gender		Nature of work	
Male	66 (60, 75)	Permanent staff	64 (54, 72)
Female	64 (56, 72)	Non-permanent staff	65 (56, 72)
Z	1.281	Z	1.378
P	0.075	P	0.045
Age (years)		Department	
20–30	64 (55, 72)	Emergency department	64 (56, 71)
31–40	65 (57, 72)	Clinical departments	65 (56, 72)
41–50	64 (54, 72)	Medical Technology departments	62 (54, 71)
≥51	64 (54.72)	Administrative departments	61 (51.70)
H	10.550	H	25.727
P	0.014	P	<0.001
Monthly income		Administrative position	
Below 3,000	65 (57, 72)	Department director/deputy director	60.5 (47.5, 72)
3,000–6,000	64 (54, 72)	Head nurse/nurse manager	63 (53, 71)
Over 6,000	70 (60, 80)	General medical staff	65 (56, 72)
H	34.752	H	16.689
P	<0.001	P	<0.001
Years of work experience (years)		Professional title	
≤5	64 (55, 72)	Junior	65 (56, 72)
6–10	64 (56, 72)	Intermediate	64 (55.5, 72)
11–20	65 (57, 72)	Associate senior	64 (53, 72)
≥21	64 (53, 72)	Full professor	52.5 (50, 72.5)
H	9.604	Other	64 (55, 72)
P	0.022	H	7.836
Marital status		P	0.098
Unmarried	64 (55, 72)	Highest education level	
Married	64 (56, 72)	College degree or below	64 (56, 72)
Divorced or widowed	64 (54, 72)	Bachelor’s degree and above	65 (56, 72)
H	0.574	Z	0.693
P	0.750	P	0.723
Number of children		Hobbies and interests	
No children	64 (55, 72)	None	66 (58, 74)
One child	64 (55, 72)	1–2 items	64 (54, 71)
Second child and beyond	65 (56, 72)	Many	66 (56, 72)
H	3.499	H	79.014
P	0.174	P	<0.001

### 3.4 Correlation analysis between job satisfaction and burnout

This study employed Spearman’s correlation analysis, revealing that job satisfaction and its various sub-dimensions were negatively correlated with occupational burnout, emotional exhaustion, and depersonalization. The ranges of correlation coefficients are −0.438 to −0.491 and −0.371 to −0.421, respectively. Indicates that higher job satisfaction correlates with lower levels of emotional exhaustion and depersonalization; it is positively correlated with reduced personal sense of accomplishment, with a correlation coefficient ranging from 0.259 to 0.310. That is, the higher the job satisfaction, the less pronounced the reduction in personal sense of accomplishment. The work satisfaction dimensions of Nurses at designated infectious disease hospitals showed a significant negative correlation with the total scores of all burnout dimensions, with statistically significant associations observed in all cases (*p* < 0.01). See Table 4.

**Table 4:** Correlation analysis of job satisfaction and burnout.

Variables	Burnout	Emotional exhaustion	Reduced personal accomplishment	Depersonalization
Job satisfaction	−0.151**	−0.491**	0.302**	−0.421**
Intrinsic satisfaction	−0.132**	−0.473**	0.310**	−0.420**
External satisfaction	−0.153**	−0.464**	0.259**	−0.371**
General satisfaction	−0.136**	−0.438**	0.298**	−0.398**

**Significant at the 0.01 level (two-tailed).

### 3.5 Multifactorial analysis of job satisfaction

The sample size for this regression analysis was 6,963 cases, with the total job satisfaction score as the dependent variable. Include variables with *p* < 0.05 from the univariate analysis—gender, professional title, administrative position, nature of work, monthly income, years of service, hobbies and interests, and occupational burnout—in the multiple linear regression. For details on the coding schemes for each variable and the reference group settings, please refer to the notes in Table 3. The results of the model assumption checks indicate that the variance inflation factor (VIF) for all variables is less than 5, suggesting no significant multicollinearity issues and satisfying the fundamental assumptions for multiple linear regression. The overall model fit is satisfactory, with *R*^2^ = 0.347 and *F* = 43.743 (*p* < 0.001), indicating that the eight variables collectively explain 34.7% of the variance in nurses’ job satisfaction. The regression results indicate: Compared with male caregivers, female caregivers reported higher overall job satisfaction (B = 4.044, 95% CI: 1.557–6.532); compared to junior-level professional titles, promotion in professional titles is negatively correlated with job satisfaction (B = −0.447, 95% CI: CI: −0.869 to −0.025); compared to ordinary medical staff, department directors/deputy directors exhibited a positive correlation in job satisfaction (B = 3.507, 95% CI: 2.199–4.815); the job satisfaction of formally employed staff significantly exceeded that of non-staff hired personnel (B = 1.183, 95% CI: 0.065–2.300); and monthly income increase is positively correlated with job satisfaction (B = 3.918, 95% CI: 3.242–4.594). Compared to those with ≤5 years of work experience, increased years of work experience were negatively correlated with overall job satisfaction (B = −1.076, 95% CI: −1.490 to −0.663); compared to those with virtually no hobbies or interests, nurses who have hobbies or interests consistently report higher overall job satisfaction (B = 1.936, 95% CI: 1.340–2.533). Higher burnout scores were negatively correlated with overall job satisfaction (B = −0.093, 95% CI: −0.114 to −0.071). See Table 5.

**Table 5:** Multiple linear regression analysis of job satisfaction influencing factors.

Variables	Unstandardized regression coefficient	SE	Standardized regression coefficient	*t*-value	*p*-value	95% CI	
Constant	60.756	3.077		19.747	<0.001	54.725	66.787
Gender	4.044	1.269	0.038	3.187	0.002	1.557	6.532
Professional title	−0.447	0.215	−0.026	−2.076	0.038	−0.869	−0.025
Administrative position	3.507	0.667	0.070	5.256	<0.001	2.199	4.815
Nature of work	1.183	0.570	0.030	2.075	0.038	0.065	2.300
Monthly income	3.918	0.345	0.154	11.357	<0.001	3.242	4.594
Years of work experience	−1.076	0.211	−0.070	−5.106	<0.001	−1.490	−0.663
Hobbies and interests	1.936	0.304	0.075	6.365	<0.001	1.340	2.533
Burnout score	−0.093	0.011	−0.101	−8.527	<0.001	−0.114	−0.071

The dependent variable is job satisfaction. Burnout scores are continuous variables. Gender and Nature of Work are dichotomous variables, with “male” and “non-permanent staff” serving as reference categories, respectively. Professional Title, Administrative Position, Years of Work Experience, and Hobbies and Interests are ordinal categorical variables; after setting dummy variables, the reference categories are “junior professional title,” “general medical staff,” “≤5 years,” and “basically none” respectively. A positive B coefficient indicates significantly higher job satisfaction compared with the corresponding reference category, while a negative B coefficient indicates significantly lower job satisfaction.

## 4 Discussion

### 4.1 Occupational burnout

An overall burnout score of 64 was found in this study, which polled 6,963 nurses at hospitals with specified infectious diseases (56, 72). Scores between 50 and 69 on the Maslach Burnout Inventory (MBI) indicate mild burnout, in accordance with established domestic classification standards (2, 18). This range was exactly where the study’s sample scores fell, indicating an intermediate level. This result is consistent with the findings of a Chinese tertiary hospital (21). Senior-level nurses’ burnout scores [52.5 (50, 72.50) points] were substantially lower than junior-level staff members’ [65 (56, 72) points] when viewed from the standpoint of professional title. This result supports the observation that “in four hospitals in Hubei, junior-level nurses experienced higher levels of emotional exhaustion and depersonalization than senior-level nurses” (22). The underlying reason is that entry-level workers are vulnerable to psychological tiredness due to the tremendous strain of executing fundamental nursing activities, keeping an eye on patient circumstances, and interacting with family members. Due to their varied professional positions and wealth of experience, people with senior professional titles have higher benefits in psychological resource regulation and show lower levels of burnout (23). Group differences analysis shows that those between the ages of 31 and 40, clinical department nurses, and non-permanent staff workers have comparatively greater levels of professional burnout. Regarding job characteristics, a study on occupational burnout among operating room nurses discovered (24) that differences in pay, benefits, and job security lead to lower occupational security for nonpermanent staff members, who also experience more emotional exhaustion and dehumanizing tendencies. Feng Jingjing et al. (25) discovered that, when looking at department type, clinical departments typically scored higher on emotional tiredness than teaching or administrative departments. Among them, time pressure, patient critical condition, and workload in frontline clinical departments are the main causes of occupational burnout. This has to do with the large number of patients and complicated circumstances in clinical departments, which put nurses under a lot of stress for extended periods of time. In terms of age-specific traits, Tomaszewska Katarzyna et al. discovered (26) that caregivers between the ages of 31 and 40 are in the core phase of juggling the demands of both their careers and their families, in addition to dealing with pressures associated with professional advancement. Compared to other age groups, there were higher scores for emotional weariness, depersonalization, and decreased personal accomplishment. According to Liu Qing’s findings that “high-income groups scored higher on burnout indicators than low-income groups,” nurses who made more than 6,000 yuan a month had the highest burnout scores [70 (60, 80) points] (27). Burnout may result from increased work intensity and expectations of duty that accompany higher income. The greatest burnout scores [65 (57, 72) points] were obtained by nurses with 11–20 years of experience, suggesting that they are in a “bottleneck period” in their career progression. Increased levels of burnout are a result of the combined impact of extended workloads and psychological strains (28). In line with research showing that American physicians “exhibited significantly higher burnout levels among those lacking hobbies compared to those with active hobbies,” caregivers who had few or no hobbies also scored higher on burnout measures (29). This implies that by reducing work-related stress and controlling emotional states, hobbies can lower the risk of burnout. This study confirmed that among nurses at designated infectious disease hospitals, occupational burnout is substantially correlated with a number of parameters, including professional title, staffing type, department assignment, age, salary level, years of service, and personal interests. While highlighting the distinctive features of burnout-associated factors within the high-risk, high-workload context of infectious disease nursing, the patterns of influence are consistent with findings from analogous studies in the nursing field. This establishes the foundation for further research into the fundamental mechanisms involved.

### 4.2 Correlation analysis between job satisfaction and burnout

This study also demonstrates a substantial negative correlation between occupational burnout and nurses’ job satisfaction at designated infectious disease hospitals (13, 14). This conclusion is consistent with research from other departments and geographical areas, such as nurses in COVID-19 wards (16), mental health care professionals in South Africa (31) and rural healthcare workers in Iran (30).and established among a range of healthcare professionals, such as physicians and midwives (31, 32). This underscores the prevalent trend in the healthcare sector that job satisfaction decreases with increasing levels of burnout. According to a dimension-specific study, the depersonalization and emotional tiredness aspects of occupational burnout were negatively correlated with each aspect of job satisfaction: By undermining their feeling of professional worth and destroying interpersonal relationships, emotional weariness and depersonalization drastically lower nurses’ job satisfaction (33), and both have a reciprocal detrimental impact on satisfaction. There is a favorable correlation between satisfaction and one’s own sense of accomplishment (34). High success can buffer stress, dramatically lower turnover intentions, and serve as a primary positive driver for improving healthcare professionals’ job happiness, according to a related survey of junior nurses in tertiary general hospitals (35). Overall, across various cultural contexts and survey settings, the detrimental impacts of emotional weariness and depersonalization, as well as the good effect of personal accomplishment, are consistent. Only a small number of covariates, including gender, show contextual heterogeneity in the strength of their influence (36).

### 4.3 Regression analysis of factors influencing job satisfaction

According to this survey, nurses at designated infectious disease hospitals had an above-average total job satisfaction score of 73 (61, 80). This is consistent with Wang Xiaohong et al.’s findings of nurses working in public health-related roles (37). This highlights the distinctiveness of infectious disease nursing roles and demonstrates the group’s basic understanding of the work. Gender, administrative position, work type, professional title, years of service, and hobbies are all important characteristics that affect job happiness, according to regression study. According to this survey, female caregivers were more satisfied with their jobs than their male counterparts. Li’s et al. (38) surveys of nurses working in China’s tertiary hospitals found similar results. In line with the large percentage of women and better occupational fit seen in the infectious disease nursing area in this study, the study also shows that female nurses exhibit greater professional identity and work fit. However, according to a survey of international literature (34) the effect of gender on nurses’ job satisfaction is frequently negligible. This discrepancy implies that gender factors do not have an absolute influence. To better understand its effect on job satisfaction, a thorough analysis that takes into account regional industry demographics, job characteristics, and the depth of the literature is required. Additionally, this finding is clearly consistent with the two types of literature’s varying research contexts.

General medical personnel had the lowest work satisfaction among administrative positions. Using general medical staff as the reference group, this regression study revealed a substantial positive association between department directors’ and deputy directors’ job satisfaction. There are several nursing studies that support this conclusion: The crucial importance of decision-making autonomy is confirmed by a review by Huang Jing et al., which shows that department heads, who have more decision-making authority and a stronger sense of value realization, reported satisfaction scores 8.3 points higher than regular medical staff (39). The mediating effect of value feedback in managerial roles is highlighted by studies by Lü et al. (40) and Sun et al. (41) that show that head nurses’ sense of professional accomplishment from their management functions significantly increases their satisfaction when compared to regular nurses. Disparities in administrative roles lead to an imbalance of authority, responsibility, and resources, which is the main cause of low satisfaction among regular medical staff: According to Wang Hongwei et al., their daily clinical burden is 1.8 times more than that of managerial positions, and professional development pathways are less clear (42). Its deficiencies in welfare protections and resource acquisition are also supported by Huang et al. (39). Thus, it is evident that variations in administrative roles often affect job satisfaction through task balance, value realization, decision-making authority, and resource allocation. Higher positions offer more resources, autonomy, and a balanced workload, all of which contribute to increased job satisfaction. On the other hand, because of the heavy workload and lack of autonomy that come with their jobs, regular medical employees are less satisfied.

Compared to non-permanent staff people, permanent staff members show noticeably greater levels of job satisfaction. This result is consistent with Wang et al.’s (15) finding that permanent nurses are substantially more satisfied than non-permanent nurses. Notably, there is a notable disparity in job satisfaction between non-permanent nurses and permanent nurses, despite their shared perception of professional importance. According to a comparative study by Chen et al. (43), permanent nurses outperformed non-permanent nurses in areas including institutional policy support and remuneration and benefits. Hard rewards and welfare continue to be the primary differentiating factor that worsens the satisfaction gap, even when both options perform equally on soft metrics like career value and doctor-patient interactions. The significantly lower job satisfaction scores of non-permanent staff nurses were likewise corroborated by a study conducted among 33 tertiary cancer hospitals (44). Their professional uneasiness is further exacerbated by the difficulties of low pay or lengthy work hours, which lowers their satisfaction levels. Fundamentally, nurses with permanent positions tend to have better pay and benefits, higher work stability, and fewer turnovers. The lack of job stability, low pay, and inadequate benefits are common problems faced by non-permanent nurses, which eventually causes a large gap in employment satisfaction between the two categories.

Using entry-level titles as a benchmark, this study discovered a substantial negative correlation between job satisfaction and advancement in professional titles. The conclusion drawn by Xia et al. (45) that satisfaction levels among nurses with greater professional titles rise with rank is not supported by this finding. Even though professional titles are frequently seen as favorable markers of career progression, in some industries, such primary healthcare and medical care, the high workload and stress of these roles may outweigh this benefit. According to a survey conducted in Shanghai among community oral public health professionals, years of service, professional title, and age all showed a strong negative correlation with all measures of job satisfaction (46). The main explanation is that more complicated patient situations and more management duties are associated with higher professional designations. The results of this study are in line with another study on family doctors that indicated a negative relationship between job satisfaction and professional title (13). This conclusion is further supported by research by Zhao (47) and others, which shows that mid-to-senior level nurses place more weight on higher-level demands like career advancement and work environment, whereas entry-level nurses prioritize basic needs like pay. The basic requirements of both categories are still not being sufficiently satisfied under the current conditions, which immediately contributes to a decline in satisfaction when professional titles increase. In particular, mid-level nurses had the lowest levels of satisfaction in the infectious disease hospital setting that was the subject of this study. This results from their dual roles as the main employees in their departments, where they must deal with a heavy workload in critical care for patients with infectious diseases and few prospects for promotion. Senior-level employees also have to respond to public health situations and manage departmental quality control, which adds to their workload and mental strain. Since they have just recently joined the company, junior employees manage comparatively simple duties with more realistic expectations, which leads to considerably greater levels of satisfaction. This discrepancy indicates that professional titles affect job satisfaction through a pressure-demand pathway and reflects the particular influences found in specialized medical contexts.

Using nurses with less than 5 years of experience as the reference group, this study discovered a substantial negative correlation between overall job satisfaction and years of service. It is hypothesized that this disagreement may be caused by variations in survey regions, hospital management systems, and compensation and benefits systems across different research, as this finding contradicts the findings of Li et al. (48). The distribution characteristics of nursing work satisfaction revealed that nurses with longer tenure had greater levels, while those with medium tenure had lower levels, which is consistent with the findings reported by Li et al. (49). This demonstrates the intricacy and contextual heterogeneity of the relationship between years of service and nurse satisfaction. According to a poll conducted among British nurses, nurses’ job satisfaction progressively decreases as their years of service increase (50). The main cause is the tendency for occupational burnout to accumulate over time as a result of long-term nursing practice. High workloads are a natural part of nurse jobs in infectious disease facilities. Over extended periods of practice, these elements, along with occupational exposure, isolation protocols, chronic physical strain, and psychological stress, gradually cause nurses to become physically and mentally exhausted. This has a direct negative impact on overall job satisfaction.

The effects of structural elements on the professional attitudes of nursing personnel, including years of service, professional titles, and administrative roles. It is challenging for people to independently alter these factors in the short term because they are rather stable. However, reducing professional burnout depends on both institutional changes and things that people may actively change. This study shows that interests and hobbies, which people are free to select, can have a big impact on job happiness. In line with findings from several studies, this study discovered that nursing staff members who had interests and hobbies reported far higher job satisfaction than those who did not. Table 3 shows that nonpermanent staff nurses had slightly higher burnout levels (65 [56,72]) than permanent staff nurses (64 [54,72]). This implies that additional psychological stresses are imposed by the job instability linked to nonpermanent status. In this context, hobbies play a particularly important psychological compensating role, providing people with a source of fulfillment separate from their professional identities. A psychological spillover effect is the underlying mechanism of this favorable connection. According to a foreign study on Iranian nurses (51), nurses’ confidence and self-efficacy in managing challenging nursing tasks are increased when they feel successful in non-medical pursuits like painting, athletics, or crafts. This positive psychological compensation from outside the job is especially important for contract nurses who are more likely to experience professional burnout. As a self-directed personal resource, hobbies and interests might somewhat offset burnout and feelings of professional helplessness brought on by unstable employment. Additional research indicates that the beneficial impact of hobbies is tempered by how well they complement nursing job. According to a 2022 cross-sectional study of nurses working in Grade A tertiary institutions, nurses’ intention to leave their jobs was significantly correlated with how well their hobbies and personal interests matched their nursing career (52). This suggests that role conflicts decrease and work-life harmony increases when personal interests complement rather than contradict employment. This significantly improves employment stability, professional fulfillment, and psychological friction. On the other hand, it will worsen psychological resource depletion and erode favorable work attitudes. In conclusion, occupational experiences are based on structural elements like job type and professional titles, but people can also actively regulate themselves within preexisting frameworks by their personal interests and how they relate to their jobs. This reminds nursing administrators that nurses should develop healthy interests and create positive connections between these activities and their work while improving institutional safeguards. Individual-level psychological adjustment can be a useful addition to institutional support, especially for non-permanent staff groupings.

Multiple linear regression analysis of the study’s data shows that occupational burnout is a substantial negative factor affecting nurses’ job satisfaction, whereas monthly salary is the largest positive factor. Numerous empirical research conducted both domestically and abroad have consistently verified this finding. Several survey findings offer compelling evidence about the impact of monthly income: One of the main determinants of job satisfaction is pay levels, according to a domestic survey of 1,319 nurses from ten provinces and municipalities (53). Community nurses in Yunyan District, Guiyang, Guizhou Province were the subjects of a study that also confirmed (54) that nurses earning ≥3,000 yuan per month had a 91.4% satisfaction rate, suggesting a strong positive relationship between income level and job satisfaction. A survey of 475 nurses and midwives in Polish healthcare facilities also suggests that economic income is the primary metric used to gauge job satisfaction at the international research level (55). The Job Demands-Resources (JD-R) model provides a clear explanation of the fundamental process that explains why compensation levels have a major impact on job satisfaction. By boosting people’s material and psychological resources, reducing occupational stress, and fostering a sense of professional belonging, compensation, a vital work resource, not only directly affects nurses’ quality of life and occupational security but also favorably influences overall satisfaction. This is in line with the JD-R model’s central claim that having enough job resources is the cornerstone for raising job satisfaction and reducing stress (7). Additionally, empirical research conducted both domestically and abroad has supported this mechanism: According to a domestic meta-analysis, “optimizing compensation is key to enhancing overall satisfaction, and nurses in China’s public hospitals have the lowest satisfaction scores in the welfare benefits dimension” (56). The favorable effect of salary as a key job resource on job satisfaction is further supported by an international meta-analysis that finds that “higher salaries significantly enhance healthcare workers’ job satisfaction” (57).

The JD-R model states that the primary cause of occupational burnout is an imbalance between job demands and resources. The exhaustion component of burnout is initially triggered when occupational demands, such as exposure to risks and high-intensity workloads, consistently overwhelm individuals without adequate resources to alleviate the strain. This, in turn, leads to disengagement. The largest negative predictor of job satisfaction is burnout, which has a substantial detrimental impact on it by reducing psychological resources, professional identity, and work engagement. This mechanism has been validated by all pertinent investigations. Depersonalization (*β* = −0.298, *p* < 0.01) and low sense of accomplishment (*β* = −0.382, *p* < 0.001), for example, are characteristics of burnout that negatively predict job satisfaction among hospital administrative personnel by lowering perceived job worth (58); The connection between job satisfaction and total burnout among mental health system employees was −0.604 (*p* < 0.001). The degree of psychological energy depletion and satisfaction decreases with increasing burnout severity (59). Among primary healthcare workers, satisfaction levels decreased by approximately 59% and 76% among those experiencing mild/moderate and severe burnout, respectively, compared to those without burnout (both *p* < 0.001), highlighting a clear dose–response relationship (60); a study of nurses at tertiary hospitals in Heilongjiang Province also indicated that while organizational commitment and satisfaction can inversely mitigate burnout, burnout remains the core driver of declining satisfaction (61). All things considered, the JD-R model offers a cohesive theoretical framework for how these three aspects interact. While burnout, a result of an imbalance between demand and resources, negatively limits job satisfaction by depleting resources and escalating disengagement, compensation, as a job resource, positively empowers job satisfaction. The outcomes of this investigation are even more credible because theoretical and empirical evidence are combined.

Additionally, this study has some limitations: the cross-sectional design makes it impossible to establish clear causal relationships between variables; the sampling was limited to specific regions, which limited the conclusions’ geographical representativeness and generalizability; and the use of self-reported data may introduce subjective biases. However, the results of this investigation have important applications. They offer empirical support for building risk-oriented remuneration structures, maximizing occupational security and compensation equity, and providing tailored intervention techniques for high-risk populations. Additionally, they offer scientific references for reducing burnout, increasing personal fulfillment, stabilizing the nursing workforce, and raising the standard of healthcare services for infectious diseases.

### 4.4 Strategies and recommendations for nursing management

The following specific nursing management techniques and suggestions are put forth in light of the study’s findings in order to reduce nurse burnout, improve job satisfaction, maintain the nursing workforce, and maximize the standard of nursing management. (1) Strengthen material assistance for high-risk groups by optimizing the career security and compensation system. Higher financial investment should be focused on improving the base pay of frontline nurses, non-regular staff, and nurses with lesser professional titles, as this study found that monthly income is the largest positive factor impacting nurses’ job satisfaction. Put in place a performance-based pay structure that correlates salary to position and gives greater incentive for exceptional work. This method links pay to performance results, workload, and occupational dangers. It seeks to reduce occupational stress, increase job satisfaction, improve pertinent welfare systems, improve non-permanent staff’s sense of job stability and belonging, and close the wage and benefit gap with regular employees. (62, 63). (2) By creating a thorough support network, concentrate on reducing burnout and promoting professional advancement. Clinical workload should be decreased for the high-risk occupational burnout categories found in this study by implementing flexible scheduling and hiring more staff. These high-risk groups should receive stress management training and psychological therapies. At the same time, we will create a mentorship program that offers individualized career planning assistance, expanding career growth opportunities for nurses. This program attempts to decrease stress, improve professional competencies, and assist nurses in overcoming developmental barriers (64). (3) To attain customized precision management, improve psychological support and individualized adaption. In order to relieve psychological stress, lower internal conflict, fortify professional identity, and improve personal-career alignment, urge nurses to take use of their interests and hobbies on a personal level by engaging in extracurricular activities. Implement interest-position matching management at the organizational level, offer customized services for nurses with different professional titles and years of experience, give gender-sensitive management top priority, improve risk subsidies and incentive systems, and foster a sense of professional belonging and job satisfaction.

## 5 Conclusion

In order to thoroughly examine the present levels of occupational burnout and job satisfaction among 6,963 nurses from designated infectious disease hospitals in Gansu Province, as well as the relationship between the two, this study used a cross-sectional survey approach. It determined the primary determinants of job satisfaction and came to the following findings: At Gansu Province’s designated infectious disease hospitals, nurses report above-average job satisfaction and a moderate overall level of occupational burnout. There is a strong inverse relationship between the two. The degree of job satisfaction decreases as occupational burnout increases. Occupational burnout among nurses is significantly influenced by a number of characteristics, including years of service, department affiliation, age, monthly income, professional title, job nature, and personal interests and hobbies. The high-risk group for occupational burnout includes non-staff employees, clinical department nurses, nurses aged 31–40, nurses with 11–20 years of work experience, and nurses without interests or hobbies. Key factors affecting nurses’ job happiness include gender, managerial position, employment status, professional title, years of service, personal interests and hobbies, monthly salary, and occupational burnout. The strongest negative predictor is occupational burnout, while the strongest positive predictor is monthly income. Among non-regular employees and general medical staff, job satisfaction is comparatively low. Additionally, because of the high-risk and high-workload nature of their profession, nurses at designated infectious disease hospitals show different satisfaction patterns with regard to professional title progression and seniority accumulation than their colleagues at general hospitals. Due to increased effort and pressure, promotions and years of service did not significantly improve job satisfaction; rather, they exhibited a negative connection. This study adds to the empirical data on occupational mental health among infectious disease nursing professionals by validating the well accepted relationship between job satisfaction and occupational burnout in the healthcare sector. In designated infectious disease hospitals in Gansu Province and comparable areas in western China, this study offers scientific support for creating nurse burnout intervention strategies, improving nursing management practices, raising job satisfaction, and stabilizing the nursing workforce.

Simultaneously, this study has disadvantages such as cross-sectional design, self-reported data bias, and single-region sample. In order to expand the sampling scope and include objective indicators, future research might use a multicenter, prospective cohort study design. This method would provide more accurate theoretical support for nursing management practice by delving deeper into the causal relationship and underlying mechanisms between occupational burnout and job satisfaction among nurses in infectious disease hospitals.

## Data Availability

The original contributions presented in the study are included in the article/supplementary material, further inquiries can be directed to the corresponding authors.
